# Determinants of Breeding Farmers’ Safe Use of Veterinary Drugs: A Theoretical and Empirical Analysis

**DOI:** 10.3390/ijerph15102185

**Published:** 2018-10-06

**Authors:** Jianhua Wang, Chenchen Yang, Hanyu Diao

**Affiliations:** 1 School of Business, Jiangnan University, Lihudadao 1800, Wuxi 214122, China; 6170909017@stu.jiangnan.edu.cn (C.Y.); 1080116222@vip.jiangnan.edu.cn (H.D.); 2 Food Safety Research Base of Jiangsu Province, Jiangnan University, Wuxi 214122, China

**Keywords:** breeding farmers, veterinary drugs, safety behavior decision, Lewin’s behavior theory

## Abstract

As food safety has attracted the widespread attention of society, the quality safety of agricultural products has become an important part of food safety and also confronts multiple challenges. In fact, the safe use of veterinary drugs in the production process has become one of important guarantees for the quality safety of agricultural products. It’s of great significance to regulate the breeding farmers’ safe use of veterinary drugs and to create a safe and healthy production environment for agricultural products. A field survey of individual and large-scale swine breeding farmers in four typical provinces including Henan, Shandong, Jiangxi and Guizhou generated 397 questionnaires. This field survey conducted the internal and external classification of breeding farmers’ safe use of veterinary drugs and defined the breeding farmers’ safe use of veterinary drugs in the light of dosage, type and standardized operation of veterinary drugs. Based on Lewin’s behavior theory, the survey used the structural equation modeling method to systematically examine the generation path of breeding farmers’ safe use of veterinary drugs. The comprehensive analysis reveals that breeding farmers’ knowledge about veterinary drugs, the attitudes toward the government supervision and the market environment of breeding activities all exert some effects on breeding farmers’ use of veterinary drugs. Some suggestions and countermeasures for breeding farmers’ safe use of veterinary drugs are provided as follows: First, more efforts should be pumped into publicity and instruction so that breeding farmers can have a better understanding of veterinary drugs. Second, preferential policies should be formulated to encourage the breeding farmers’ participation in the industrial organizations of swine breeding farmers, and advocate the industrial organizations’ active provision of different technical trainings. Third, the communication and cooperation platform should be created among breeding farmers, slaughter and processing plants and supermarkets, the poultry insurance market should be regulated, and the insurance purchase process should be improved. Fourth, when more subsidies for harm-free and environment-friendly veterinary drugs are provided, more serious punishments should be imposed on the unsafe use of veterinary drugs to offer policy support for the breeding farmers’ standardized use of veterinary drugs.

As one of the factors of production used by breeding farmers to guarantee the healthy growth of bred animals, veterinary drugs play a tangible role in the swine breeding process. However, the current dosage, type and administration of veterinary drugs haven’t reached the required level. The calculation based on loss control model shows that the actual margin productivity of veterinary drugs is almost zero. This means that the swine breeding farms in most regions have been found to make an excessive use of veterinary drugs [1,2]. The national government promulgated the Measures for the Administration of the Prescription and Non-prescription Drugs for Veterinary Use in 2013, and the Ministry of Agriculture promulgated the Catalogue of Veterinary Prescription Drugs (Batch II) in 2016. As a matter of fact, the Ministry of Agriculture promulgates the national monitoring plan of veterinary volatile residues of animal and animal products. It’s easy to see that the state attaches much importance to the control over the volatile residues of veterinary drugs and illicit drugs.

In recent years, there have been quite a few safety accidents caused by the improper use of veterinary drugs throughout the country, such as “the removal of live fish from supermarket”, “the abuse of veterinary drugs in the chickens provided for Dicos”. These safety accidents have caused the safe use of veterinary drugs to become a public concern again. The Strategic Research Report on Status Quo, Problems and Countermeasures of Food Safety in China, a significant consulting project published by the Chinese Academy of Engineering, also pointed out that the abuse of veterinary drugs is the main source of pollution threatening food safety in China. Veterinary drugs are abused in the breeding process, and even human drugs are used in the breeding industry. As a result, human health faces potential threats. The large scale addition of antibiotics to animal feeds has drawn much concern from the industry. Therefore, the description and analysis of the actual use of veterinary drugs and the exploration into the influencing factors of decision-making conducts about safe use of veterinary drugs can provide the rational and scientific interpretation for the improper use of veterinary drugs, and the theoretical support for the government’s supervision, the related social participants’ supervision and the breeding farmers’ understanding of their personal conducts, for the ultimate purpose of reducing dosage of veterinary drugs, regulating the type of veterinary drugs used, and guaranteeing the breeding farmers’ safe operation of veterinary drugs. 

China is the leading pork producer and consumer in the world. The use of veterinary drugs in the swine breeding process has much to do with the swine quality, and can affect the consumers’ health and the market activity in the whole society. The dosage, type and administration of veterinary drugs by swine breeding farmers are affected by multiple factors. Based on these considerations, this paper mainly examines those factors that affect the breeding farmers’ safe use of veterinary drugs, and tracks the generation path of breeding farmers’ use of veterinary drugs. 

## 1. Literature Review

Regarding the use of veterinary drugs in the swine industry production process, the research of domestic and foreign scholars has a different focus. Foreign scholars give great prominence to the research on the quality safety of animal products, but because the agricultural production technologies in Europe and America enjoyed early and high-level development, the foreign scholars pay more attention to technical use, consumers’ intentions and efficiency in the use of veterinary drugs. The study by Feder et al. showed that the education background could affect the producers’ intention to use the new technologies [3]. In a study on information-based supervisory technologies for the safety of animal products, Petersen et al. conducted an analysis of such variables as slaughtering information, consulting service information and health management systems [4]. When it comes to the party responsible for pork safety, Erdem found in the study that both producers and consumers believed that the other party should have a stronger sense of responsibility [5]. The foreign studies of the cognitive characteristics of swine breeders also deserve consideration. Valeeva et al. pointed out that the swine breeders’ understanding of the importance of swine quality safety and their perception of disease risks affected their use of veterinary drugs [6]. Garforth et al. discovered in their study that breeding farmers’ use of veterinary drugs could be influenced by a series of internal factors like their education and risk preference [7]. In the studies on those factors that affect the breeding farmers’ use of veterinary drugs, some foreign scholars suggested the classification of these factors according to internal and external factors. Lynne and Rola included the variants of social and psychological conducts in the economic decision-making process and concluded that the breeding farmers’ decision-making conducts were affected by their own attitudes and targets, and also by endogenous variants like personal characteristics and exogenous variants like social conditions [8]. Lewin’s behavior theory was used not merely in production behaviors, but in consumers’ behaviors as well. Tong and Li used Lewin’s behavior theory as the theoretical basis in the study on those factors that influence the demand for personal mobile telecommunication services, and the structural equation modeling was used to create the study framework for endogenous and exogenous variables. This study could offer some insights [9].

Domestic scholars pay more attention to studies on breeding farmers’ intentions and conduct as well as the factors influencing their use of veterinary drugs. Meanwhile, an increasing number of domestic studies has explored the regulatory work, policies, government organization, social coordination and market factors when the national situations of China are considered. In the studies on breeding farmers’ conducts involved in the use of veterinary drugs and the swine breeding, Wang et al. mainly classified the safety risks in the production process of swine supply chain into internal safety risks, external safety risks and systematic safety risks, and finally concluded that the limited policy awareness, insufficient integration of industrial chain and hard access to information were the main sources of internal and external risks, and the systematic risks mainly came from diseases and natural disasters [10]. The swine breeding farmers’ personal characteristics including gender, age, education, breeding history and safety awareness about the volatile residues of veterinary drugs can all exert some significant influence on the breeding farmers’ use of veterinary drugs [11]. For the effects of breeding farmers’ personal characteristics, production characteristics, and cognitive level during withdrawal period of veterinary drugs on their quality control conducts, Chang and other scholars came to a similar conclusion. In addition to internal factors, the change in the organizational pattern of breeding farmers can have a comprehensive effect on the control of production quality. The tight organizational form can help breeding farmers to develop the positive quality control habit and the membership of cooperatives can contribute much to the breeding farmers’ conducts [12]. Pu et al. used the measurement model to analyze and identify two characteristics of those breeding farmers in the use of restricted veterinary drugs. The internal characteristics included poor education and large numbers of breeding farmers, but the external characteristics included less rigorous quarantine at breeding sites [13]. Besides the identification and analysis of those factors that affect the breeding farmers’ different uses of veterinary drugs in the above-stated studies, there are different opinions about the improper use of veterinary drugs. Most scholars studied the single misuse of veterinary drugs, but it proves more objective and specific to classify the breeding farmers’ misuse of veterinary drugs according to the logics behind the use of veterinary drugs. Therefore, the breeding farmers’ misuse of veterinary drugs can be classified into excessive use of veterinary drugs, use of human medicines for domestic animals and fowls, and unsafe operation in the use of veterinary drugs.

The examination of the above literature shows that scholars’ studies on breeding farmers’ use of veterinary drugs are mainly focused on the internal and external factors, but the factors covered in their studies were less comprehensive and representative. Therefore, this paper will, based on the previous studies, take internal and external factors into consideration and explore the generation path of breeding farmers’ use of veterinary drugs under the influence of internal and external factors.

## 2. Theoretical Basis and Modeling

The joint effects of individuals and scenarios on behaviors have long been evidenced by the personality and social psychology. Since the 20th century, some psychologists and sociologists have performed in-depth studies on the laws of human behavior in an attempt to gain insights into the causes behind complicated behaviors. The U.S. social psychologist Kurt Lewin’s research findings have been put into wide use [14]. Based on many analyses and experiments, Lewin proposed the famous “field” theory to explore the behavioral research method, which is also known as “Lewin behavior theory”. This model summarizes all the essential factors that influence human behaviors and concludes that the behaviors are affected by two mutually independent and interactive factors, namely individuals and environments (social and non-social). Before the “field” theory, the psychoanalysis method and Skinner’s behavioral pattern theory were used to analyze individual conducts, but they have shortcoming to different degrees. The first method merely focuses on individual influences, but neglects the environmental influences, but the second method puts individuals exclusively under the control of environment, but neglects the influence of personality structure [15]. Therefore, the theoretical models based on “Lewin’s behavior theory” are highly generalized and widely applicable, so they reap the broad recognition.

The model of Lewin’s behavior theory is detailed as follows:

(1) B=f(P, E) 

In Equation (1), *P* (Personal) stands for a person’s internal characteristics and conditions in the initial model. It can be such variables as *P*_1_, *P*_2_, *P*_3_, …… to represent all the physiological and psychological factors that constitute the internal conditions like physiological ability, character and attitude. *E* (Environment) stands for a person’s external environment in the initial model. It can use variables such as *E*_1_, *E*_2_, *E*_3_, …… to represent all the environmental factors like the natural environment, social environment and economic environment. In this model, human behaviors can be considered the result of joint action of human and environmental factors. Further analysis also reveals that human behavioral form, direction and intensity are all restricted by internal factors and external environments. This paper examines the swine breeding farmers’ use of veterinary drugs and the influencing factors. The study in this field focuses on the utilization efficiency of veterinary drugs and also explores the effects of breeding farmers’ psychological factors, but external conditions and the environment play a tangible effect role in breeding farmers’ decisions. Lewin’s behavior theory can offer the theoretical support for the comprehensive analysis of internal and external factors. When the breeding farmers’ cognition is analyzed, it is necessary to take environmental factors into consideration, expanding the scope of the survey and optimizing the thinking structure.

### 2.1. Utilization Behavior of Veterinary Drugs

Scholars disagree on the ways to measure the breeding farmers’ utilization behavior of veterinary drugs. A general analysis showed that the measurement methods are mainly divided into single indicator-based measurements, multiple indicators-based measurements, and comprehensive indicator system-based measurements. Because some important variables may be neglected by the single indicator-based measurements and more personal considerations may be included in the comprehensive indicator-based measurements, scholars prefer to use the multiple indicators-based measurements. Liu’s study measured whether breeding farmers’ decisions about their ecological behaviors involve the strict use of feeds, drugs and additives, whether the water fowls’ excrements are properly disposed of, and whether the ecological breeding is used [16]. In studies on cow quality safety behaviors, the characteristics of safety conducts are divided into the safety management of cows’ breeding methods, hygiene sand safety management in the breeding process, and safety management in the milking process [17]. Based on the previous studies, this paper classified the utilization behavior of veterinary drugs as follows: whether veterinary drugs are administered according to the drug description on most occasions; whether veterinary drugs are administered according to the drug description in case of urgent epidemic situations; whether human drugs are applied to swine; whether human drugs are used instead of veterinary drugs; whether swine will face diseases or other risks; which veterinary drugs breeding farmers are willing to use. The periodic testing and entrusted monitoring of veterinary volatile residues in animals and animal products are conducted to check the utilization results of veterinary drugs.

For such a question as whether veterinary drugs are administered according to the drug description, the related studies show that the breeding farmers consider the factors like the quality of veterinary drugs, prevention and control results of epidemic situation and inaccurate indication of dosage, and often make an excessive use of veterinary drugs in the actual production process. This behavior tends to cause the serious excess of volatile residues of veterinary drugs, so product quality and human health are seriously threatened [18,19,20]. Some problems are found with the proper use of drugs by kind when it comes to the veterinary drugs under banned or restricted use and the use of human medicines for veterinary purposes. The unsafe conduct of breeding farmers will cause a series of undesirable consequences. Guo came to the conclusion in a study on the testing methods of volatile residues in the swine veterinary drugs under banned or restricted use in Gonggang, Jiangxi Province that some swine farms used more kinds of veterinary drugs than the national level [21]. The volatile residues of veterinary drugs caused by the use of illicit drugs will gradually accumulate inside human bodies and even threaten human kidneys and livers, respiratory systems, circulation system and gastrointestinal system [22]. After some knowledge is gained about the damages caused by the excessive use of veterinary drugs and the use of veterinary drugs of unauthorized kinds, the volatile residues of veterinary drugs are also the important factors that threaten the safe use of veterinary drugs. The withdrawal period of veterinary drugs can ensure to some degree that the volatile residues of veterinary drugs reach the required level. The withdrawal period means the interval between the discontinued use of veterinary drugs in animals and the approved slaughtering or introduction of animal products (milk and eggs) to market. The veterinary drugs in animal bodies can’t vanish unless they have gone through such processes as absorption, metabolism and excretion. Some breeding farmers’ non-compliance with the regulations within the withdrawal period can pose some potential threats to the consumers, including deformations, mutations, cancers, hormones, allergic reactions and environmental pollution [23].

Therefore, the regular testing of volatile residues of veterinary drugs is an important way to regulate the use of veterinary drugs. Based on the retrieval and analysis of safe uses of different veterinary drugs, the study uses the multiple indicators-based measurement to track breeding farmers’ use of veterinary drugs, and provides the optimization path for regulating the use of veterinary drugs at different levels.

### 2.2. Internal Factors

Based on theoretical analysis and literature review, the internal factors that affect the use of veterinary drugs can be divided into a person’s internal characteristics and conditions. As far as the use of veterinary drugs is concerned, breeding farmers’ intentions to use safe veterinary drugs are affected by gender, age and breeding history [24,25]. Liu’s study revealed that female breeding farmers use veterinary drugs in a more cautious and standardized way [26]. Some scholars have also mentioned that the educational background of breeding farmers in the mariculture field also affected their use of veterinary drugs [27].

Besides the individual factors, the breeding farmers’ knowledge about veterinary drugs is also an important factor that affects the use of veterinary drugs. According to Wu’s research, the more awareness breeding farmers have about the proper use of veterinary drugs, the more standardized use of veterinary drugs they will have [28]. Those breeding famers with stronger awareness about the correct use of veterinary drugs and the damages caused by volatile veterinary drug residues are more likely to have the right conduct and attitude and more willing to display positive quality safety conduct [29]. Liu’s study came to the opposite conclusion that breeding farmers with more knowledge about the regulations on restricted drugs (the regulations on withdrawal period and utilization quantity of veterinary drugs) tend to have more standardized use of veterinary drugs. The reason is that the better-informed breeding farmers have a stronger motive to guarantee the health of livestock and poultry by using veterinary drugs. When swine isn’t allowed to get slaughtered, breeding farmers would rather venture to make improper use of veterinary drugs, but want to face no losses. In the meantime, he also concluded in this study that, the more rigorous testing of swine quality safety a swine buyer conducts, the more standardized use of veterinary drugs a breeding farmer may have [26]. Pu’s study showed that, the more frequent use is made of the restricted veterinary drugs, the stronger awareness that the discontinuation of harmful drugs is very essential for human health. This unexpected conclusion also matches the practical conclusion in the domestic breeding environment. This has something to do with the practice of “overtly agreeing and covertly opposing” in everyday life [13].

When it comes to the studies on the breeding farmers’ awareness of government policies, Wu’s study found that the breeding farmers’ knowledge about the related laws and regulations as well as the punishments have some limited effects upon their use of veterinary drugs [28]. This paper treats individual factors, household factors and business characteristics as the control variables, which will not be included in assumption, but will be examined in the subsequent study. Based on the above-stated literature review, the following assumptions are made:

**H1.** *Breeding farmers’ knowledge about veterinary drugs, awareness of the importance of safe use of veterinary drugs and awareness of the government’s supervision will exert a significant effect on the breeding farmers’ use of veterinary drugs.*

**H2.** *Breeding farmers’ knowledge about veterinary drugs, awareness of the importance of safe use of veterinary drugs and awareness of the government’s supervision will exert a significant effect on the breeding farmers’ use of human drugs for veterinary purpose.*

**H3.** *Breeding farmers’ knowledge about veterinary drugs, awareness of the importance of safe use of veterinary drugs and awareness of the government’s supervision will exert a significant effect on the standardized operation of veterinary drugs.*

### 2.3. External Factors

Swine breeding farmers’ use of veterinary drugs is also affected by the external environment. Zhou included some related factors in his study and concluded that the external environment (government regulations and market factors) could indirectly influence conducts through human behavioral cognition [30]. Some scholars have also stated that the root causes of vegetable growers’ quality safety conducts were the breeding farmers’ professionalism and participation in cooperatives [31]. Liu discovered in a study that the swine breeding farmers’ use of veterinary drugs could get more regulated when a tighter oral and written model was generated through the vertical agreement and an integrated sales model was created [26]. In Britain’s studies on the breeding farmers’ use of antibiotic drugs, scholars like Coyne found that the management of agricultural system can affect the farmers’ use of antibiotic drugs [32]. For market factors, some scholars also pointed out that the farm owners in Holland may consider the market gains when the use of veterinary drugs is taken into consideration. The use of veterinary drugs could also be influenced by the market-based incentive measures [33].

In addition to market factors, the government regulations are also the important factors that can affect the swine breeding farmers’ use of veterinary drugs. Wu mentioned in a study that government supervision and acctions, like a larger punishment for breaching the regulations, can effectively mitigate the negative effects of swine breeding farmers’ use of antibiotic veterinary drugs [28]. Sun et al. discovered in the study that when more rigorous testing was conducted on the slaughtered swine at the swine’s place of origin, the breeding farms/farmers had stronger incentives to implement satisfactory quality safety conduct [29]. Visschers et al. found in the study that farmers were very concerned about any changes in the government’s laws and policies, and that this factor can largely affect their use of veterinary drugs [34]. What’s more, the government’s training of farmers on the correct use of pesticides can also help reduce their use of illicit pesticides [35]. Several foreign scholars’ investigations and studies of those livestock and poultry products processing companies revealed that market can play a more incentive role than the government [36]. Based on the literature review, the following assumptions about external factors are made:

**H4.** *Market factors and government regulations will exert a significant effect on the breeding farmers’ use of veterinary drugs.*

**H5.** *Market factors and government regulations will exert a significant effect on the breeding farmers’ use of human drugs for veterinary purpose.*

**H6.** *Market factors and government regulations will exert a significant effect on the breeding farmers’ standardized operation of veterinary drugs.*

**Figure 1:**
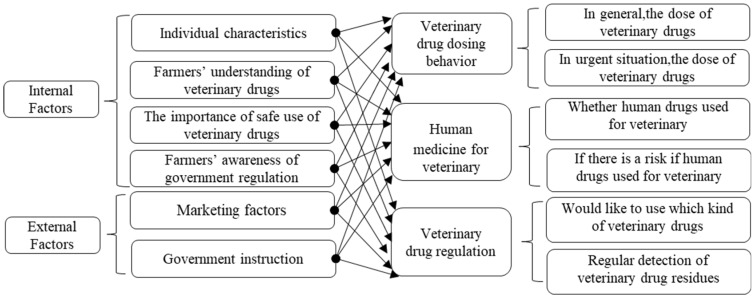
Behavioral model of safe veterinary drug use by farmers.

Based on the above theoretical analysis and model analysis, the paper aims to explore the breeding farmers’ path selection in terms of the use of veterinary drugs after the internal and external factors influencing the use of veterinary rugs are classified and identified, and to establish the well-organized market mechanism and the well-prepared government regulations that standardize the use of veterinary drugs, so as to guarantee the breeding farmers’ safe production, lay basis for the consumers’ swine consumption and create a positive environment for food safety (see Figure 1 for the model).

## 3. Research Method

The Structural Equation Model, also known as latent variable model, is used in this paper to study the influencing factors of veterinary drug use behavior of farmers. Based on the covariance matrix of variables, the model analyzes the relationship between variables to better identify the relationship between latent variables, which is regarded as a more universal measurement model. In the structural equation model, the latent variable that is difficult to directly observe is represented by a manifest variable that can be directly observed and processed, and the factor analysis and the path analysis are combined. Unlike traditional statistical methods, the Structural Equation Model considers and deals with multiple dependent variables while introducing latent variables. Factor relationships are measured when the factor structure is estimated, and the fitness of the whole model is evaluated [37,38,39].

The Structural Equation Model consists of a measurement model and a structural model. The expressions are shown in the following three linear equations [40]:

(2) $$x=\Lambda_{x}\xi +\delta \;$$

(3) $$\;x=\Lambda_{y}\eta +\varepsilon \;$$

Equations (2) and (3) are expressions of the measurement model, indicating the relationship between the latent variable and the observed variable. Among them, *x* is the measurement variable of exogenous latent variable; *y* is the measurement variable of endogenous latent variable; *ξ* is the exogenous latent variable; ᴧ*_x_* is the correlation matrix of exogenous latent variable and observation variable; ᴧ*_y_* is the correlation matrix of endogenous latent variable and observation variable:

(4)  η=Bη+Γξ+ζ 

Equation (4) is an expression of the structural model that reflects the relationship between latent variables. Among them, *B* is the relationship between endogenous latent variables; *Г* is the influence of exogenous latent variables on endogenous latent variables; *ζ* is the residual term of the structural equation, reflecting the unexplained part of the equation.

## 4. Data Source and Sample Feature Analysis

### 4.1. Data Source

The research data of decision-making on the safe use of veterinary drugs by farmers was obtained from the food safety research base of Jiangnan University and the field research of retail and large-scale farmers in four typical provinces of Henan, Shandong, Jiangxi and Guizhou. Since Henan and Shandong are both big pig farming provinces, the investigation was first carried out in these two provinces. The research team went to the survey area from January to February 2017 to investigate the use of veterinary drugs by pig farmers in 2016 for the purpose of grasping the basic situation of pig farming nationwide. In order to ensure better representativeness of the sample data, the research team subsequently released and collected questionnaires in Jiangxi and Guizhou so as to increase the sample size and optimize the sample structure.

When deciding the sample size, the research done by Hou and Huo was referred to, and it was decided that the sample size of the first stage should include at least six sample units [41]. After determining the sample size, counties and cities with typical breeding scale and quality were selected by using the specific sampling method where the probability and scale are proportional. The counties and cities selected are: Kaifeng City, Heze City, Linyi City, Shangrao City, Luoyang City, Xinzheng City, Bijie City, Anyang County. According to the calculation formula determined by the number of farmer households in the second stage, the number of the sample size should be at least 275. In this paper, stratified random sampling is adopted as the principle of overall sampling. The specific sampling process is that a certain number of sample cities (counties) are selected first, and then several villages (towns) are selected from each sample city (counties). For the selection of sample villages, according to the proportion of aquaculture, random sampling is carried out in the high, medium and low levels to ensure that the samples can be randomly determined in the sample village. After selecting the sample village, the investigators contact the village cadres or relevant personnel who grasp the overall situation. Finally, farmers were selected in each sample village to participate in the survey.

Before investigating the selected samples, the research team carried out a small-scale pre-survey in Xinzheng City (Henan Province) and revised the questions that were unclear and ambiguous or made people unwilling to answer so as to ensure the validity of the questionnaire. For the members of the research group, professional research experts were arranged to conduct pre-research training to ensure the authenticity and reliability of the data. In order to ensure the correctness of the content filling in by the correspondents and their response rate, the researchers used a combination of interviews and questionnaires, and the respondents were family members who played a decisive role in pig farming. In the survey, 480 questionnaires were actually collected, questionnaires with missing key data and untrue answers were excluded and thus 397 valid questionnaires were finally obtained. The variables involved in the empirical analysis model and their descriptive statistics are shown in Table 1.

### 4.2. Descriptive Statistical Analysis of Control Variables

According to the theories and assumptions mentioned above, this paper introduces three classes of 10 control variables. The meanings, assignments and corresponding descriptive statistics of the variables are shown in Table 1.

It can be seen from Table 1 that the respondents were mostly male, with an average age of 50–60 years old. The education level is 6–9 years, which is not high. This index is consistent with the age. Therefore, the older respondents are less educated. The average number of farm households engaged in pig farming was stable for 5–10 years, and there are more farmers engaged in part-time work besides pig farming. In terms of the family characteristics, the average number of family number is more than four, with an average annual income of 40,000 to 8000 yuan, accounting for over 50% of the total household income. The average scale of pig farming is between 50 and 100 heads. The average breeding models are controlled between individual retailers and large professional households. It can be seen that scale of the pig farming is small, and there are fewer large-scale farmers such as professional households and family farms.

### 4.3. Analysis of the Current Status of Veterinary Drug Use Behavior

After understanding the basic characteristics of the farmers, descriptive statistics on the behavior of the veterinary drugs used by the farmers were obtained to know the current status of the veterinary drug use by farmers. Six explanatory variables were introduced. The meanings, assignments, and corresponding descriptive statistics of the variables can be seen in Table 2.

The non-standard use of veterinary drugs can be divided into three categories, namely, the use of veterinary drug dosage behavior, human medicine veterinary behavior, veterinary drug standard operation behavior. The overall situation of the use of veterinary drugs in farms is general, and there are still irregular behaviors of veterinary drug use. It can be seen from the Table 2 that the behavior of veterinary drug use is divided into different situations. Among them, the proportion of the farmers who strictly follow the instructions is 57.18%. those who use less than twice as much as the instructions 36.78%, who use nearly twice as much as the instruction 6.05%. In case of the emergent epidemic situations, the proportion of the farmers who strictly follow the instruction to dispense drugs is 27.71%, those who use less than twice as much as the instructions 55.16%. It is clear that in an emergent situation, the number of people using veterinary drugs less than twice as many as required by the instructions is greatly increased. In the case of the human medicine for veterinary use, 39.29% of the farmers said that they had never used human medicine on pigs; 27.96% of the farmers said that they often use human medicine for pigs; 12.59% of the farmers thought that human medicine for veterinary use would not bring any risks to the pigs; and 41.06% of farmers were aware of the harms of the human medicines for veterinary use. In terms of the operational standards of veterinary drugs, 57.93% of the farmers were only willing to use common veterinary drugs and had no intention of using pollution-free or green veterinary drugs. About 71.04% of the farmers would carry out regular veterinary drug volatile residue detection or entrusted monitoring in animal and animal products. According to the comprehensive analysis of veterinary drug use behavior, 20% to 30% of the farmers will adopt irregular veterinary drug use behavior in the process of the breeding, including excessive use of veterinary drugs, human medicine for veterinary use, and non-standard operational behaviors of veterinary drug use.

## 5. Study on Influencing Factors for Decision Making of Safe Veterinary Drug Use Behaviors of Farmers

### 5.1. Model Building

**Figure 2:**
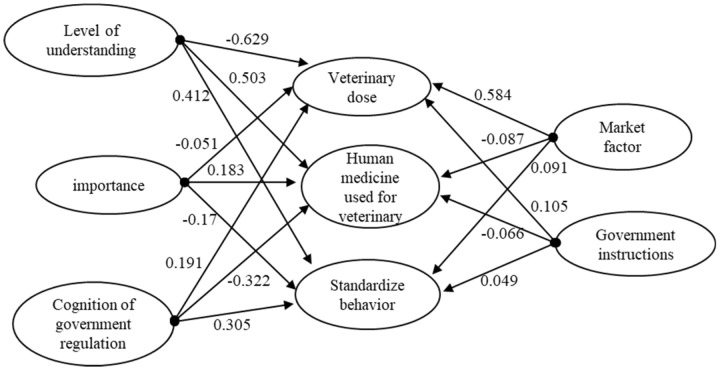
Path diagram of SEM (Structural Equation Modeling).

This paper presents a structural equation model as shown in Figure 2. The model takes the influencing factors of veterinary drug use behavior as endogenous latent variables. The knowledge of veterinary drugs of farmers, the importance of the farmers for safe veterinary drug use, and the cognitive characteristics of farmers on government supervision, market factors and government rules and regulations are exogenous latent variables (Table 3).

### 5.2. Exploratory Factor Analysis and Reliability and Validity Test

This paper used the statistical software packages SPSS 22.0 (International Business Machines Corporation, Armonk City, NY, USA) and AMOS 21.0 (International Business Machines Corporation, Armonk City, NY,) to conduct a series of factor analyses of the collected data. Among them, SPSS 22.0 was mainly used to carry out the tests of the exploratory factors analysis and reliability, while AMOS 21.0 was mainly used for the validity tests of the conceptual models. 

#### 5.2.1. Analysis of the Exploratory Factors

The exploratory factor analysis of the variables is first performed using the software, and the output rotation factor loading matrix is shown in Table 4. According to the results of software operation, the KMO (Kaiser-Meyer-Olkin) value of the total scale is 0.817, and the KMO value of the subscale is more than 0.7; the Bartlett sphere chi-square test is significant, and the Sig value of the significance coefficient is 0.000, less than 0.05. In addition, the standard factor load coefficients of each measurement variable are basically above 0.5, and the standard is greater than 0.5, indicating that the sample data is suitable for factor analysis.

#### 5.2.2. Reliability Test

The reliability test is used to verify the reliability of the design questionnaire. The larger the test coefficient, the greater the reliability of the measurement. In this paper, Cronbach’s Alpha coefficient was used as the reliability of the test variables. SPSS22.0 software was used to analyze the reliability of 20 observation variables of five latent variables, such as farmer’s understanding of veterinary drugs, the importance of safe veterinary drugs, the awareness of government supervision, market factors, government regulation and other five latent variables. After many calculations, inappropriate measurement variables were eliminated (see Table 4). It is generally considered that Cronbach’s Alpha coefficient is not credible in the range of 0.6–0.65, and it is the minimum acceptable value in the range of 0.65–0.70, which is quite good in the range of 0.70–0.80, and the reliability of the scale is very good in the interval of 0.80–0.90 [42]. After analyzing the questionnaire variables, it was found that the Cronbach’s Alpha coefficient of the questionnaire total scale was 0.785, and the Cronbach’s Alpha coefficient of each subscale was above 0.65. Both of them passed the reliability test, indicating that the internal consistency between the variables was good.

#### 5.2.3. Validity Test

Validity tests include convergence validity and discriminant validity. The convergence validity is expressed by C.R (Critical ratio) value and AVE (average variance extracted) value. When C.R value is above 0.7 and AVE value above 0.6., the convergence validity is good. The validity of the sample data was tested by AMOS21.0 statistical software. The results are shown in Table 4. The C.R and AVE values of each kind of potential variables meet the criteria, indicating that the validity of each potential variable is good and the questionnaire designed is ideal.

The test for discriminant validity is shown in Table 5. In order to achieve acceptable discriminant validity, the results of the inter-group relationships of potential variables in the measurement model are required to be lower than their intra-group relationships. The relationship matrix is used to measure the inter-group relationship of latent variables. The results show that the root of the AVE, that is, the diagonal value is smaller than the inter-group coefficient of the underlying potential variable, indicating that the data has excellent discriminant validity [43].

### 5.3. Model Test Results and Analysis

#### 5.3.1. Parameter Test and Fitting Evaluation

AMOS 22.0 software (International Business Machines Corporation, Armonk City, NY, USA) was used to carry out regression analysis on five latent variables and their observed variables such as degree of understanding, importance, awareness of policy supervision, market factors and government regulation. The path map and path coefficients shown in Figure 2 were obtained. 

It can be seen from Table 6 that overall goodness of the structural equation model of this study is relatively good, and all the evaluation indexes have reached the ideal state.

For the behavior of veterinary drugs, the standardized path coefficients of the five latent variables such as the degree of understanding of veterinary drugs, the awareness of the importance of safe veterinary drugs, the perception of government regulation, market factors, and government regulation are −0.629, −0.051, 0.191, 0.584, 0.105, respectively. The standardized path coefficient of producer’s understanding of veterinary drugs was the largest, and it passed the test at the 1% significance level. Therefore, the variable had the most obvious influence on the behavior of veterinary drug dosage, and the effect was negative. Because when assigning the behavior of veterinary drug dosage, the behavior from the normative ono to the non-normative one increases in turn. So, the negative correlation indicates that the more the farmers understand the veterinary drugs, the less likely the farmers are to use veterinary drugs. The producer’s cognition of the importance of safe veterinary drugs has not passed the test, and the impact on the medicinal quantity behavior of the farmers is not significant. Farmers’ perceptions of government regulation and market factors passed the test at 5% and 1% significance levels, respectively. The degree of influence was that market factors come before farmers’ perception of government regulation. The government regulatory environment in which the farmers are located has no significant effect on the medicinal quantity behavior of the farmers. It can be seen that some of the ideas in hypothesis 1 and hypothesis 4 are verified.

For the behavior of human medicine for veterinary use, the standardized path coefficients of five latent variables, namely, knowledge of veterinary drugs, importance of safety veterinary drugs, government supervision, market factors and government regulation are 0.503, 0.183, −0.322, −0.087 and −0.066, respectively. Among them, the standardized path coefficient of the producer’s understanding of veterinary drugs is the largest, then the variable has the most significant effect on the veterinary behavior of the farmers. The correlation was positive, indicating that the greater the farmers’ understanding of veterinary drugs, the greater the possibility of farmers refusing to take human medicinal veterinary behavior. The producer’s awareness of the importance of safe veterinary drugs has a significant effect on the farmer’s medicinal veterinary behavior at the 1% significant level. Farmers’ perception of government regulation passed the test at a 1% significant level. The market factors and the regulatory environment of the farmers have no significant impact on their medicinal and veterinary behavior. To sum up, the assumption two is valid, and the fifth refused.

For the standard operation behavior, the standardized path coefficients of the five latent variables such as the producer’s understanding of veterinary drugs, the awareness of the importance of safe veterinary drugs, the perception of government supervision, market factors, and government regulation are 0.412, −0.17, 0.305, 0.091 and 0.049, respectively. Among them, the standardized path coefficient of the producer’s understanding of veterinary drugs is the largest and passed the test at the 5% significance level. The variable has the most significant effect on the standard operation behavior of the farmers. The correlation is positive, indicating that the farmers are veterinary drugs. The greater the level of understanding, the greater the likelihood that farmers will regulate the use of veterinary drugs. Producers’ awareness of the importance of safe veterinary drugs had no significant effect on farmers’ standardized use of veterinary drugs. Farmers’ perception of government regulation was tested at a 1% significant level. Market factors and government regulation environment have no significant impact on farmers’ standardized use of veterinary drugs. It can be assumed that assumption 3 is partially verified, and 6 is not valid.

#### 5.3.2. Factor Load Analysis of Measurement Models

The factor load coefficient reflects the influence of observed variables on latent variables, as shown in Table 7. Among the latent variables of farmers’ understanding of veterinary drugs, LD5 was the biggest influencing factor, and the standardized path coefficient was 0.845, which was significant at 1% statistical level. This indicated that the more the producers knew about the list of veterinary drugs prohibited by the state, the more the farmers knew about veterinary drugs. Among the farmers’ awareness of the importance of safe veterinary drugs, the biggest influencing factor is IS4. The standardized path coefficient is 0.872, and it is significant at the 1% statistical level. This indicates that when the reason that the safe veterinary drugs are beneficial for the consumers’ health is of great significance in the farmers’ choice of safe veterinary drugs, the farmers have greater awareness of the importance of the safe veterinary drug use. CG3 is the most important factor affecting farmers’ understanding of government regulation, and the standardized path coefficient is 0.909, which is significant at 1% of the statistical level. This indicates that farmers’ awareness of government regulation will increase with the increase of the likelihood of local government supervision on the safe use of veterinary drugs. Among the market factors, the biggest one is MF1, and the standardized path coefficient is 0.800, which is significant at 1% of the statistical level. It indicates that whether the producer joins the pig industrialization organization is the most influential factor in the external market factors. The more often farmers join the pig farming industry organizations, the more favorable conditions they are likely to obtain. The biggest influencing factor in the government regulation of farmers is GI2. The standardized path coefficient is 0.485, and it is significant at the 1% statistical level. This indicates the mandatory immunization vaccine provided by the local government is the most influential factor in the government regulation of farmers. In other words, the government’s regular provision of mandatory immunization vaccines will reduce the farmers’ concerns about the pig epidemic and the risk of disease and promote the adoption of standardized veterinary drug use by farmers.

## 6. Conclusions and Inspirations

Based on the sample data of 397 farmers in four provinces, this paper uses a structural equation model to study the behavior of veterinary drug use of farmers, further divides the behavior of veterinary drug use of farmers, and clarifies the main factors affecting the different behavior of veterinary drug use of farmers. The results showed that farmers’ understanding of veterinary drugs, farmers’ awareness of government supervision and market factors all have an impact on veterinary drug consumption behavior. Among them, farmers’ understanding of veterinary drugs has the greatest impact on the behavior of veterinary drug consumption, showing a negative correlation at the 1% significance level. This reflects that farmers’ understanding of veterinary drugs is the most critical factor affecting the behavior of veterinary drug consumption. Among them, it is also the farmers’ knowledge of the veterinary drugs that has the greatest impact on the behavior of human medicine for veterinary use. Farmers’ understanding of veterinary drugs, farmers’ awareness of the importance of safe veterinary drugs, and farmers’ awareness of government supervision have an impact on farmers’ normative operation behavior. Farmers’ understanding of veterinary drugs has the greatest impact on farmers’ normative operation behavior. For the three kinds of veterinary drug use behavior, farmers’ understanding of veterinary drugs is the most important factor.

In response to the abovementioned conclusions, this paper proposes the following countermeasures and suggestions. First, strengthen publicity and guidance to improve the understanding of producers on veterinary drugs, including the use of veterinary drugs, drug withdrawal period, knowledge of pig disease and other aspects. Government departments should increase publicity efforts so that farmers can understand the harms of unsafe veterinary drugs and the benefits of safe veterinary drugs. Second, encourage farmers to participate in the industrialization of pig breeding through the formulation of preferential policies, and encourage industrial organizations to actively carry out various technical training to create a good market environment for producers. Third, build a communication and cooperation platform for farmers, slaughtering and processing enterprises and supermarkets, regulate the poultry insurance market, optimize the insurance process, and regulate producer behavior through the power of market organization. Fourth, while increasing the subsidies for pollution-free and green veterinary drugs, strengthen the punishment for the unsafe use of veterinary drugs, and provide policy support for farmers to regulate the use of drugs.

## Figures and Tables

**Table 1:** Descriptive statistics of control variables.

Variables		Variable Definition	Mean Value	SD
Personal characteristics	Gender	Female = 0; Male = 1	0.75	0.43
	Age	20–30 years old = 1; 30–40 years old = 2; 40–50 years old = 3; 50–60 years old = 4; 60 years old or above = 5	3.23	0.91
	Education	Less than 6 years = 1; 6–9 years = 2; 9–12 years = 3; 12–15years = 4; 12 years or more = 5	1.94	0.75
	Years working for pig farm	Less than 1 year = 1; 1–2 years = 2; 2–5 years = 3; 5–10 years = 4; more than 10 years = 5	3.45	0.99
	Whether full time	no = 0; yes = 1	0.60	0.49
Family characteristics	People in family	1 person = 1; 2 persons = 2; 3 persons = 3 4 persons = 4; 5 persons or more = 5	4.33	0.73
	Annual family income	Less than or equal to 40,000 yuan = 1; 40,001–80,000 yuan = 2; 80,001–12,000 = 3; 120,001–160,000 = 4; more than 160,000 yuan = 5	1.78	0.90
	Percent of Farming income in annual family income	30% and less than 30% = 1; 31%~50% = 2; 51%~80% = 3; 81% or above = 4	2.09	1.03
Operating characteristics	Scale of farming pigs	Less than 50 = 1; 50–100 = 2; 100–200 = 3; 200–500 = 4; 500 or more = 5	1.56	0.98
	Farming style	Individual = 1; Professional = 2; Family Farm = 3; others = 4	1.35	0.81

Note: Scale of farming pigs: the amount of livestock on hand, including piglets, boar, sows, etc.

**Table 2:** Descriptive statistics of the interpreted variables.

Variable Classification	Measured Variable	Variable Assignment	Number of Samples	Percentage
Veterinary drug dosing behavior	Generally, are they prepared according to the instructions of veterinary drugs	Follow the instructions strictly = 1	227	57.18%
		Less than 1 times more than the instructions require = 2	146	36.78%
		Nearly 2 times more than the instructions require = 3	24	6.05%
		More than 2 times more as much as the instructions requires = 4	0	0
	If the epidemic is urgent, is it configured according to the veterinary drug instructions?	Follow the instructions strictly = 1	110	27.71%
		Less than 1 times more than the instructions require = 2	219	55.16%
		Nearly 2 times more than the instructions require = 3	53	13.35%
		More than 2 times more as much as the instructions requires = 4	15	3.78%
Human medicine and animal use behavior	Whether to use human drugs on pigs	Yes = 1	54	13.60%
		Generally = 2	57	14.36%
		Sometimes = 3	130	32.75%
		Never = 4	156	39.29%
	Whether the use of human drugs instead of veterinary drugs will bring disease or other risks to pigs	no = 1	50	12.59%
		unclear = 2	163	41.06%
		yes = 3	184	46.35%
Veterinary drugs regulate operational behavior	Would like to use which of the following veterinary drugs	Regular veterinary drugs = 1	230	57.93%
		Pollution-free veterinary drug = 2	135	34.01%
		Green veterinary drugs = 3	32	8.06%
	Regularly carry out veterinary drug volatile residue testing or commissioned monitoring in animal and animal products to check the effect of veterinary drugs	Strongly disagree = 1	16	4.03%
		Disagree = 2	18	4.53%
		Not sure = 3	81	20.40%
		Agree = 4	231	58.19%
		Strongly agree = 5	51	12.85%

**Table 3:** Structural equation variables affecting the behavior of farmers’ veterinary drugs.

Potential Variable (Code)	Measured Variable (Code)	Variable Assignment
Farmers’ understanding of veterinary drugs (LD)	Do you know the drug withdrawal period for the use of veterinary drugs? (LD1)	Don’t know at all = 1; don’t know = 2; generally understand = 3; better understand = 4; very understand = 5
	Do you know about veterinary drug volatile residues (LD2)	Don’t know at all = 1; don’t know = 2; generally understand = 3; better understand = 4; very understand = 5
	Do you know about safe veterinary drugs (LD3)	Don’t know at all = 1; don’t know = 2; generally understand = 3; better understand = 4; very understand = 5
	Do you know the Regulations on the management of Veterinary Drugs (LD4)	Don’t know at all = 1; Don’t know very well = 2; general understand = 3; well understand = 4
	Do you know the list of banned drugs for veterinary drugs that are banned by the government? (LD5)	Don’t know at all = 1; Don’t know very well = 2; general understand = 3; well understand = 4
The importance of safe use of veterinary drugs (IS)	The reason for the purchase of pigs is to choose the importance of using safe veterinary drugs. (IS1)	Not important = 1; not very important = 2; generally important = 3; more important = 4; very important = 5
	The reason for safety and risk-free is the importance of choosing safe veterinary drugs. (IS2)	Not important = 1; not very important = 2; generally important = 3; more important = 4; very important = 5
	The reason why pork quality is good is the importance of choosing safe veterinary drugs. (IS3)	Not important = 1; not very important = 2; generally important = 3; more important = 4; very important = 5
	The reason for the health of consumers is the importance of choosing safe veterinary drugs. (IS4)	Not important = 1; not very important = 2; generally important = 3; more important = 4; very important = 5
Farmers’ perception of government regulation (CG)	How easy is it to obtain information on legal policies and practices in pig farming (CG1)	Very difficult = 1; more difficult = 2; general = 3; more convenient = 4; very convenient = 5
	Your evaluation of the level of local government’s epidemic prevention (CG2)	Very dissatisfied = 1; dissatisfied = 2; generally satisfied = 3; not very satisfied = 4; very satisfied = 5
	Your evaluation of the local government’s supervision of the safe use of veterinary drugs (CG3)	Very dissatisfied = 1; dissatisfied = 2; generally satisfied = 3; not very satisfied = 4; very satisfied = 5
	How often do you think the local government regulates the safe use of veterinary drugs? (CG4)	Very low = 1; low = 2; general = 3; high = 4; very high = 5
Marketing Factors (MF)	Whether to join the pig breeding industrialization organization (MF1)	No = 1; occasionally = 2; often = 3
	Does the industrial organization provide technical training? (MF2)	No = 1; occasionally = 2; often = 3
	Whether it has signed sales contracts with slaughtering and processing enterprises and supermarkets (MF3)	No = 1; occasionally = 2; often = 3
	Whether to buy poultry insurance (MF4)	No = 1; occasionally = 2; often = 3
Government Instructions (GI)	Whether the quarantine department will go to your farm to carry out on-site inspection and quarantine when the pigs are out? (GI1)	No = 1; occasionally = 2; often = 3
	Does the local government provide mandatory immunization? (GI2)	No = 1; occasionally = 2; often = 3
	Does the local government punish the unsafe use of veterinary drugs? (GI3)	No = 1; occasionally = 2; often = 3

Note: LD, Learn degree; IS, Importance of Safe ; CG, Cognition of Government.

**Table 4:** Variable exploratory factor analysis, reliability and validity test results.

Potential Variable (Code)	Measured Variable (Code)	Cronbach’α	Factor Loading	KMO Sample Measure	C.R	AVE
Farmers’ understanding of veterinary drugs (LD)	Do you know the drug withdrawal period for the use of veterinary drugs? (LD1)	0.860	0.814	0.822	0.864	0.661
	Do you know about veterinary drug volatile residues (LD2)		0.802			
	Do you understand safe veterinary drugs? (LD3)		0.655			
	Do you know the Regulations on the Administration of Veterinary Drugs? (LD4)		0.718			
	Do you know the list of banned drugs for veterinary drugs that are banned by the state? (LD5)		0.687			
The importance of safe veterinary drug use (IS)	The reason for the purchase of pigs is to choose the importance of using safe veterinary drugs. (IS1)	0.873	0.795	0.820	0.878	0.643
	The reason for safety and risk-free is the importance of choosing safe veterinary drugs. (IS2)		0.862			
	The reason why pork quality is good is the importance of choosing safe veterinary drugs. (IS3)		0.868			
	The reason for the health of consumers is the importance of choosing safe veterinary drugs. (IS4)		0.857			
Farmers’ perception of government regulation (CG)	How easy is it to obtain information on legal policies and practices in pig farming (CG1)	0.839	0.657	0.741	0.840	0.678
	Your evaluation of the level of local government’s epidemic prevention (CG2)		0.848			
	How often do you think the local government regulates the safe use of veterinary drugs? (CG3)		0.869			
	Your evaluation of the local government’s supervision of the safe use of veterinary drugs (CG4)		0.687			
Market factor (MF)	Whether to join the pig breeding industrialization organization (MF1)	0.699	0.809	0.650	0.718	0.600
	Does the industrial organization provide technical training? (MF2)		0.684			
	Whether it has signed sales contracts with slaughtering and processing enterprises and supermarkets (MF3)		0.656			
	Whether to buy poultry insurance (MF4)		0.529			
Government instructions (GI)	Whether the local government has provided subsidies for pollution-free veterinary drugs or green veterinary drugs (GI1)	0.652	0.570	0.679	0.712	0.676
	Does the local government provide a mandatory immunization vaccine? (GI2)		0.563			
	Does the local government punish the unsafe use of veterinary drugs? (GI3)		0.765			
Veterinary drug dose use behavior	In general, is it configured according to the veterinary drug instructions?	-	0.519	0.753	-	-
	If the epidemic is urgent, is it configured according to the veterinary drug instructions?		0.554			
Veterinary drug type use behavior	Never used human medicine on pigs	-	0.792	0.711	-	-
	Whether the use of human drugs instead of veterinary drugs will bring disease or other risks to pigs		0.682			
Veterinary drug specification use behavior	Which of the following veterinary drugs are willing to use	-	0.704	0.701	-	-
	Regularly carry out veterinary drug volatile residue testing or commissioned monitoring in animal and animal products to check the effect of veterinary drugs					

**Table 5:** Discriminant validity of latent variables.

Potential Variables	Level of Understanding	Importance	Cognition of Government Regulation	Market Factors	Government Regulation
level of understanding	0.813				
Importance	0.095	0.801			
cognition of government regulation	0.566	−0.32	0.823		
market factors	0.337	−0.099	0.205	0.775	
government instructions	0.587	0.089	0.605	0.243	0.822

**Table 6:** Overall fitness evaluation standard and fitting evaluation result of structural equation model.

Fit Index	Specific Indicators	Suggested Value	Observed Value	Conclusion
Absolute fit index	X^2^/df	<2	1.521	ideal
	RMR	<0.05	0.077	closely
	GFI	>0.9	0.903	ideal
	RMSEA	<0.05	0.036	ideal
Relative fit index	NFI	>0.9	0.923	ideal
	TLI	>0.9	0.952	ideal
	CFI	>0.9	0.964	ideal
Information index	AIC	The smaller the better	674.493	ideal
	CAIC	The smaller the better	1267.582	ideal

Notes: RMR: Root mean square residual; GFI: Goodness of fit index; RMSEA: Root mean square error of approximation; NFI: Normed fit index; TLI: Tucker-Lewis index; CFI: Comparative fit index; AIC: Akaike information criterion; CAIC: Consistent Akaike information criterion.

**Table 7:** Structural coefficient table of structural equation model. (Please define the symbols “**” and “***” and explain the abbreviations we marked.)

Path			Parameter Estimate Value	SE (Std error)	Critical Ratio	Standardized Path Coefficients
Structural equation						
Veterinary dose	←	LD	−0.345	0.08	−4.322	−0.629 ***
Veterinary dose	←	IS	−0.016	0.017	−0.942	−0.051
Veterinary dose	←	CG	0.119	0.049	2.414	0.191 **
Veterinary dose	←	MF	0.206	0.045	4.567	0.584 ***
Veterinary dose	←	GI	0.028	0.026	1.093	0.105
Human drugs for veterinary purpose	←	LD	0.474	0.135	3.502	0.503 ***
Human drugs for veterinary purpose	←	IS	0.096	0.037	2.6	0.183 ***
Human drugs for veterinary purpose	←	CG	−0.345	0.113	−3.064	−0.322 ***
Human drugs for veterinary purpose	←	MF	−0.053	0.044	−1.211	−0.087
Human drugs for veterinary purpose	←	GI	−0.031	0.047	−0.654	−0.066
Normative behavior	←	LD	0.263	0.105	2.496	0.412 **
Normative behavior	←	IS	−0.06	0.036	−1.671	−0.17
Normative behavior	←	CG	0.221	0.077	2.884	0.305 ***
Normative behavior	←	MF	0.037	0.039	0.955	0.091
Normative behavior	←	GI	0.015	0.043	0.357	0.049
Measuring equation						
LD1	←	LD	1.000	−	−	0.629
LD2	←	LD	0.957	0.070	13.624	0.597 ***
LD3	←	LD	1.129	0.101	11.231	0.674 ***
LD4	←	LD	1.641	0.130	12.584	0.797 ***
LD5	←	LD	1.784	0.135	13.182	0.845 ***
IS1	←	IS	1.000	−	−	0.749
IS2	←	IS	0.986	0.064	15.336	0.811 ***
IS3	←	IS	0.898	0.059	15.297	0.811 ***
IS4	←	IS	1.049	0.068	15.311	0.872 ***
CG1	←	CG	1.000	−	−	0.473
CG2	←	CG	1.978	0.194	10.183	0.879 ***
CG3	←	CG	2.143	0.210	10.219	0.909 ***
CG4	←	CG	1.368	0.136	10.071	0.605 ***
MF1	←	MF	1.000	−	−	0.584
MF2	←	MF	1.859	0.187	9.940	0.800 ***
MF3	←	MF	0.574	0.078	7.319	0.356 ***
MF4	←	MF	1.317	0.153	8.599	0.567 ***
GI1	←	GI	1.000	−	−	0.851
GI2	←	GI	1.013	0.124	8.151	0.485
GI3	←	GI	0.713	0.112	6.378	0.353
DW1	←	DW	1.000	−	−	0.422
DW2	←	DW	2.465	0.466	5.292	0.845 ***
UHM1	←	UHM	1.000	−	−	0.429
UHM1	←	UHM	1.261	0.293	4.312	0.818 ***
SB1	←	SB	1.000	−	−	0.469
SB2	←	SB	1.432	0.266	5.384	0.485 ***

Note: * p < 0.1; ** p < 0.05; *** p < 0.01. LD,Learn degree ; IS, Importance of Safe ; CG,Cognition of Government ; MF,Market factor ; GI,Government instructions ; DW, Dose weight ; UHM, Use of human medicine ; SB, Standardize behavior.
